# Epidemiology and prognostic implications of panic disorder and generalized anxiety disorder in patients with coronary artery disease: rationale and design for a longitudinal cohort study

**DOI:** 10.1186/s12872-021-01848-3

**Published:** 2021-01-12

**Authors:** Guillaume Foldes-Busque, Clermont E. Dionne, Stéphane Turcotte, Phillip J. Tully, Marie-Andrée Tremblay, Paul Poirier, Isabelle Denis

**Affiliations:** 1 School of Psychology, Université Laval, 2325 rue des Bibliothèques, bureau 1018, Québec, QC G1V 0A6 Canada; 2 Research Center of the Centre Intégré de Santé et de Services Sociaux de Chaudière-Appalaches, Lévis, QC Canada; 3 Research Center of the Quebec Heart and Lung Institute, Québec, QC Canada; 4 Hôpital du Saint-Sacrement, Québec, QC Canada; 5 Department of Rehabilitation, Faculty of Medicine, Université Laval, Québec, QC Canada; 6 School of Medicine, The University of Adelaide, Adelaide, Australia; 7 Faculty of Pharmacy, Université Laval, Québec, QC Canada

**Keywords:** Anxiety disorders, Panic disorder, Generalized anxiety disorder, Coronary artery disease, Prognosis, Revascularization, Adverse cardiac events

## Abstract

**Background:**

Anxiety is associated with poorer prognosis in patients with coronary artery disease (CAD). Due to their severity and chronic course, anxiety disorders, particularly generalized anxiety disorder (GAD) and panic disorder (PD), are of considerable interest and clinical importance in this population. This study has two main objectives: (1) to estimate the prevalence and incidence of GAD and PD in patients with CAD over a 2-year period and (2) to prospectively assess the association between PD or GAD and adverse cardiac events, treatment adherence, CAD-related health behaviors, quality of life and psychological distress.

**Design/Method:**

This is a longitudinal cohort study in which 3610 participants will be recruited following a CAD-related revascularization procedure. They will complete an interview and questionnaires at 5 time points over a 2-year period (baseline and follow-ups after 3, 6, 12 and 24 months). The presence of PD or GAD, adherence to recommended treatments, health behaviors, quality of life and psychological distress will be assessed at each time point. Data regarding mortality and adverse cardiac events will be collected with a combination of interviews and review of medical files.

**Discussion:**

This study will provide essential information on the prevalence and incidence of anxiety disorders in patients with CAD and on the consequences of these comorbidities. Such data is necessary in order to develop clear clinical recommendations for the management of PD and GAD in patients with CAD. This will help improve the prognosis of patients suffering from both conditions.

## Background

In Canada and in the United States, over 6% of adults aged 20 years or older live with coronary artery disease (CAD), the most common form of cardiovascular disease [1, 2]. Along with its elevated prevalence, CAD is also one of the costliest diseases in both countries, with annual direct and indirect costs reaching 219$ billions a year in the United States [2–4]. Although the incidence and mortality rates associated with CAD have decreased in the last decades, it remains one of the leading causes of mortality, hospitalization and disability-adjusted life years lost worldwide [1, 5, 6].

A variety of interventions may be used to manage the potential consequences of CAD and improve the prognosis of patients [7–9]. Given that up to 90% of the myocardial infarction risk is attributable to nine modifiable risk factors (e.g., cholesterol levels, hypertension, diabetes, smoking, obesity, physical inactivity, alcohol use, diet and psychosocial factors), comprehensive risk factor management programs and cardiac rehabilitation are essential to improve outcomes in patients with CAD [8, 10, 11]. The management of psychosocial risk factors represents a particular challenge: while decades of research have demonstrated their importance in patients with CAD, the nature and implications of the relationship between CAD and anxiety disorders is not as well understood as that of other CAD risk factors [12–17]. Although much of the research to date has focused on the role of depression, a growing number of studies suggest that anxiety may also lead to negative outcomes in this population [18–22].

### Anxiety and coronary artery disease

Elevated anxiety has been independently associated with a 36–88% increase in the risk of adverse cardiac events in patients with CAD [22–32]. However, the relationship between anxiety, CAD and CAD-related mortality remains unclear [33]. This may be partly due to the fact that anxiety, particularly anxiety disorders, remains understudied in the context of CAD [33]. One important issue is that most of the studies on this topic assessed anxiety with self-reported questionnaires [33]. This approach alone cannot be used to diagnose anxiety disorders which, due to their severity and chronic course, are more likely to have a significant impact on CAD prognosis [21, 28].

Two anxiety disorders, generalized anxiety disorder (GAD) and panic disorder (PD), are of particular interest and importance in patients with CAD as their prevalence rates among this clinical population (24 and 53% respectively), are up to 15 times higher than those in the general population [21, 33–39]. In patients with CAD, the presence of these disorders is also associated with an increased risk of major cardiac events, greater disability, higher psychological distress and lowered quality of life [21, 39–44]. Both disorders are also characterized by an increased suicidal risk and multiple and often unproductive medical consultations [45–50] as well as incident CAD and cardiac events [22, 51, 52]. Without treatment, PD and GAD have a chronic course and worsen over time, which negatively influence their prognosis and treatment response [46, 53–55].

Diagnosing anxiety disorders such as GAD and PD in patients with CAD can be challenging as there is a significant overlap in the somatic symptoms of both conditions (e.g. chest pain, dizziness, dyspnea, palpitations and tiredness) [20, 21, 33, 35, 56]. This may explain why anxiety disorders have been found to be more prevalent in the few studies in which they were diagnosed using structured interviews (the gold standard for psychiatric diagnoses) conducted by trained mental health professionals [33]. Furthermore, a single assessment point, as was used in several reviewed studies, might not be reliable in order to adequately identify pathological anxiety in patients with CAD [20–22, 24, 25, 27, 31–33]. Indeed, some authors have expressed concerns that assessing anxiety disorders shortly after a cardiac or life-threatening event may lead to false positives [28, 35]. In addition, the onset of a chronic illness such as CAD and dealing with its consequences are risk factors for the development of GAD and PD [57] and could lead to an elevated incidence of these disorders in the following months and years. Thus, further prospective studies using validated structured interviews and a robust methodology to assess the prevalence and incidence of PD and GAD in patients with CAD are needed [33].

### Possible mechanisms linking panic disorder and generalized anxiety disorder with CAD prognosis

Though still unclear, physiological and behavioral pathways have been proposed to explain how anxiety disorders and CAD influence each other and subsequently lead to poorer outcomes. Part of the association between CAD and anxiety disorders may be explained by physiological factors, such as increased inflammation [20, 21, 36, 39, 58]. Anxiety disorders are also associated with higher rates of hypertension, obesity, diabetes and dyslipidemia, which all increase the cardiovascular risk [21, 29, 59]. PD and GAD have also been linked to poorer health behaviors [20, 21, 36, 60–64]. For instance, the likelihood of alcohol use disorder, either dependence or abuse, is 79–83% higher in patients with GAD or PD than in patients without these disorders [65, 66]. These anxiety disorders are also associated with a 50–90% increased risk of daily smoking and nicotine dependence as well as low levels of physical activity [61, 63, 64, 67–70]. Furthermore, high levels of anxiety have been associated with non-attendance and non-completion of cardiac rehabilitation programs and non-adherence to cardiac medication [64, 70–75]. Consequently, another part of the association between anxiety disorders and CAD could be explained by the negative influence of these disorders on health behaviors and adherence to evidence-based risk-reducing recommendations and treatments for CAD [20, 21, 56, 64, 70, 75].

### Summary

Despite the recommendations of the American Heart Association in 2014 [18], very few studies have prospectively investigated the independent role and differential impacts of anxiety disorders on cardiovascular outcomes in patients with established CAD [33, 76]. While some studies assessed the prevalence and prognostic implications of GAD in patients with CAD, to our knowledge, no prospective study has documented the potential consequences of PD on these same outcomes.

Anxiety disorders, and more specifically PD and GAD, are associated with a wide array of unhealthy behaviors, but the prevalence and persistence of such behaviors in patients with CAD remain unknown. Moreover, no study has investigated the role of PD and GAD on enrollment, participation and adherence to cardiac rehabilitation. Finally, only one study accounted for both medical and behavioral risk factors while assessing the effects of GAD on adverse cardiac outcomes in patients with CAD [31]. This low number of studies combined with concerns regarding diagnostic accuracy in most of them severely limit the interpretation of the data available on this issue, underscoring the importance of further research on the prognostic significance of PD and GAD in patients with CAD.

### Objectives

In order to help bridge the knowledge gaps related to PD and GAD in patients with CAD, this study has two main objectives:

1. To establish the prevalence and incidence of PD and GAD in the 2 years following a CAD-related revascularization procedure.
2. To prospectively assess the association between the presence of PD or GAD and:
  - Adverse cardiac events;
  - Adherence to treatments (cardiac rehabilitation, pharmacotherapy);
  - CAD-related health behaviors;
  - Quality of life and psychological distress.

## Methods

### Design

This is a prospective cohort study in which participants will be evaluated at 5 time points: baseline, 3 months, 6 months, 1 year and 2 years. The study was registered with clinicaltrials.gov (NCT04433832).

### Participants

All adults (≥ 18 years) who undergo a CAD revascularization procedure at the coronary/cardiac surgical care unit of the Quebec Heart and Lung Institute and are fluent in French will be eligible for study participation. These patients will be recruited either onsite or following their transfer to the Hôtel-Dieu de Lévis University-Affiliated Hospital. Patients will be excluded if they present a severe communication problem or suffer from a terminal illness, a diagnosed major cognitive deficit, or any other condition that could invalidate the interview (e.g. psychotic disorder), of if they are not legally competent.

### Measures and outcomes definitions

#### Presence of PD and GAD (objective 1)

PD and GAD will be assessed at each time point using the Anxiety and Related Disorders Interview Schedule for DSM-5 (ADIS-5) [77]. This standardized interview protocol is one of the recommended measures for diagnosing anxiety disorders, including PD and GAD, in the research context [33, 78].

#### Adverse cardiac events (objective 2a)

Patients will be asked about all potential adverse events occurring during the study period with a structured medical interview developed by our team [79, 80]. All data will be confirmed by an independent review of medical records. With written consent from patients or next of kin (if deceased), records for admissions or consultations outside of the study hospital will be obtained from the hospital archives. Medical data will be extracted using a combination of a standardized medical data extraction form known for its excellent reliability (Cohen’s k = 0.72–0.91) and modules from the GRACE initial form [79–81].

Adverse events are defined as: either an acute myocardial infarction as defined by the Joint ESC/ACCF/AHA/WHF Task Force for the Universal Definition of Myocardial Infarction [82], a revascularization procedure (percutaneous coronary intervention or coronary bypass grafting), a cardiac arrest (including ventricular fibrillation) or death from a cardiovascular cause (primary cause).

The main outcome for objective 2a is the presence of any of the listed adverse events. The association between the presence of PD or GAD and each individual type of event will also be explored.

#### Adherence to treatments (objective 2b)

*General measure*: Adherence to treatments will be assessed with the Medical Outcomes Study Measures of Patient Adherence [83]. This brief structured interview, converted to a questionnaire for the purpose of this study, assesses the patients’ tendency to comply with medical recommendations and contains 2 sections (general and heart disease-specific) [83, 84]. This measure has good internal consistency (Cronbach’s α = 0.81) [83]. For the purpose of this study, the items were translated to French using Vallerand’s back-translation methodology [85].

*Medication adherence*: This parameter will be assessed using the Adherence Scale in Chronic Diseases which has good internal consistency (α = 0.74) [86–88]. This scale was translated to French using Vallerand’s back-translation methodology [85].

*Cardiac rehabilitation participation*: Patients will be asked if they participated in a formal cardiac rehabilitation program at each time point with two validated interview items [89]. Attendance will be assessed by reviewing records of the 12-week cardiac rehabilitation programs. With written consent from patients or next of kin, records of attendance to programs outside of the study hospital will be obtained from hospital archives. The overall level of adherence will be calculated with the percentage of the prescribed sessions attended during the program. Participation will be categorized as follows:

- Non-attendance: patients who did not attend a cardiac rehabilitation program.
- Completion: patients who attended at least 70% of the planned sessions [90].
- Non-completion: patients who attended less than 70% of the planned sessions [90].
- Discontinuation: patients who stopped attending cardiac rehabilitation at least 3 weeks before the end of the program.

#### Health behaviors (objective 2c)

*Level of physical activity*: This parameter will be assessed with the validated French version of the International Physical Activity Questionnaire—Short Form [91]. This data will be used to determine if patients are following the recommendations of the American Heart Association regarding exercise, which are: at least 30 min (minimum 5 days per week) of moderate intensity aerobic activity such as brisk walking [10].

*Smoking status*: This variable will be assessed using the validated self-report items developed by Statistics Canada to assess the current smoking status (non-smoking, occasional smoker, smoking every day) [92] in the 30 days preceding each time point.

*Alcohol use*: The patients’ compliance with recommendations regarding alcohol consumption (≤ 1 drink/day for women and ≤ 2 drinks/day for men) [7, 8, 10, 93, 94] will be assessed with the first 3 items of the validated French version of the Alcohol Use Disorders Identification Test [93, 94]. These items are well validated and are highly correlated with objective measures of alcohol consumption [95].

*Fruit and vegetable intake*: Patients usual consumption of fruit and vegetables will be assessed using the 6-item Fruits and Vegetables Questionnaire [96]. This brief measure is predictive of overall eating habits.

#### Quality of life and psychological distress (objective 2d)

*Health-related quality of life*: The validated French version of the 12-item Short-Form Health Survey Version 2 will be used to assess the health-related quality of life of patients [97, 98]. The physical summary score and mental summary score will be used in the current study.

*Psychological distress*: Anxiety and depressive symptoms will be measured with the validated French version of the Hospital Anxiety and Depression Scale [99, 100]. The internal consistency of the questionnaire and its 2 subscales (anxiety and depression) is well established (α = 0.68–0.93) [101]. The total score will be used as a measure of overall psychological distress in the current study. Heart-focused anxiety will be assessed with the French version of the Cardiac Anxiety Questionnaire [102, 103]. It has good internal consistency (α ≥ 0.83) and satisfactory convergent validity [102].

#### Other variables and measures

*Sociodemographic data*: Sociodemographic information, including employment status, educational level and family income (i.e. socio-economic status), will be obtained using a brief questionnaire.

*Medical comorbidities and cardiovascular risk factors for CAD*: This data will be obtained through a medical interview and review of medical records. Obesity will be assessed using body mass index (kg/m^2^), which will be computed from the reported height and weight of patients obtained during the medical interview. The Charlson Comorbidity Index will be used to summarize the patients’ medical comorbidities [104, 105].

#### Other psychosocial risk factors for CAD

- Social support will be assessed with the validated French version of the Modified Medical Outcomes Study Social Support Survey. This brief measure assesses the patients’ perceived social support and has good internal consistency (α = 0.85–0.88).
- Depression, agoraphobia, health anxiety and somatic symptom disorder will be assessed with the ADIS-5 which presents good to excellent inter-rater agreement for these psychiatric diagnoses [106].
- Post-traumatic stress disorder (PTSD) will be assessed using the French version of the PTSD Checklist for DSM-5, a 20-item self-report measure that assesses symptoms of post-traumatic stress disorder [107, 108].
- Sleep habits and sleeping problems will be assessed by using the French version of the Pittsburgh Sleep Quality Index [109, 110]. The brief self-reported questionnaire has good internal consistency (α = 0.70–0.83) and test–retest reliability [109].

### Procedures

#### Recruitment and assessment of patients

Research assistants at the recruitment site will identify potentially eligible patients by consulting medical files. They will subsequently present them the consent form, explain the research project, answer questions and, upon acceptance, administer the medical interview, the fruits and vegetables intake measure and the physical activity measure. Face-to-face or phone assessments with a trained interviewer (doctoral student in psychology) to administer the selected modules of the ADIS-5 will be scheduled onsite with the consenting patients. Patients will fill out the questionnaires right after the face-to-face interview in an electronic format with assistance from a research assistant if needed. Patients who are unable to come in for a face-to-face interview (either due to their medical condition or to distance from the recruitment site) will be offered to complete the interview by phone and to fill out the questionnaires online through the REDCap secure web-based application [111], by phone or by regular mail. For regular mail and website completion, patients will receive a follow-up call to offer assistance a week after the questionnaires are sent. All measures will be administered at all time points. Patients will be contacted  two weeks before each time point in order to schedule the interview (onsite or by phone) and questionnaire completion (electronic format, paper or by phone). Patients who are unreachable after three weeks will be considered lost to follow-up. At each time point, research personnel, blinded to the patients’ results to the ADIS-5, will review the medical records of all patients. To improve retention, patients will have a chance to win an electronic tablet for the completion of each time point (interview and questionnaires) as a compensation for their time.

#### Quality control

Assessors (psychology doctoral students who completed ≥ 180 h of academic courses and practicums focusing on clinical assessment) will receive initial training (14 h) and subsequent weekly clinical supervision. The ADIS-5 interviews will be audio-recorded to facilitate supervision and to realize inter-rater agreements on the diagnoses. Inter-rater agreements on all ADIS-5 interviews and data will be established using a random sample of 25% of all files reviewed and all ADIS-5 interviews realized at each time point. All questionnaires and interviews will be reviewed after completion and patients will be contacted if missing data are present. Finally, most of the questionnaires will be filled out electronically, reducing the risk of incorrect or missing data.

### Statistical analyses

All study data will be collected and managed using the REDCap data capture tools [111]. For objective 1, the prevalence and incidence of GAD and PD will be presented with their 95% confidence intervals for each time point. As a sensitivity analysis, the stability of baseline diagnoses of PD and GAD will be assessed at the 3-month follow-up. Diagnoses will be considered stable if less than 10% of patients experience remission (no longer meeting the diagnostic criteria for PD or GAD) between these time points. If this criterion is not fulfilled, all analyses for objective 2 will be repeated using the diagnoses established at the 3-month follow-up to identify patients with and without PD or GAD. Inter-rater agreements on ADIS-5 diagnoses will be assessed by computing Cohen’s Kappa and its 95% confidence interval.

In the context of objective 2, generalized linear mixed models (continuous outcomes) and generalized estimating equation models (dichotomous outcomes) with group X time interactions will be used to compare the evolution of both groups (with and without GAD or PD) in terms of adverse cardiac events, adherence to treatments (cardiac rehabilitation, pharmacotherapy), CAD-related health behaviors, quality of life and psychological distress All models will be adjusted for age, sex, cardiovascular risk factors, medications and medical comorbidity. Additional control variables will include, when relevant: diagnosis of depression, PTSD symptoms, previous cardiac events and interventions, health behaviors and social support. In the presence of significant effects of PD or GAD, analyses will be repeated in order to explore differences among men and women (group X sex X time).

As an exploratory measure, analyses will be repeated separately for GAD and PD. All analyses will be repeated using 3 groups (patients with PD or GAD at baseline, patients who developed PD or GAD afterwards, patients without PD or GAD at all time points) in order to explore the impact of developing GAD or PD over time.

#### Justification of sample size

The minimal sample sized required for each objective was calculated separately. The final sample size was established according to the objective requiring the highest number of participants (i.e. objective 2a, n = 2527 participants). That estimation was based on a previously reported rate of adverse cardiac events of 14.3% [22]. Considering an anticipated attrition rate of 30% after 2 years, a total of 3610 patients will have to be initially recruited. Based on the lower range of findings from studies of GAD in patients with CAD, the relative risk of adverse events in patients was conservatively estimated at 1.50 [33]. This data was used in the absence of similar studies in patients with PD.

## Discussion

PD and GAD appear to be highly prevalent in patients with CAD and their negative impact on cardiovascular, physical and psychological outcomes in this population are increasingly recognized. However, the literature on this issue is still scarce, and the available studies present considerable methodological flaws. Furthermore, very few studies accounted for the effects of traditional risk factors, treatment adherence and health behaviors while examining the impacts of PD or GAD in patients with CAD. Consequently, the prevalence of anxiety disorders and their prognostic role in patients with CAD remain unclear, and the current available literature cannot support clear clinical recommendations for the management of PD and GAD in patients with CAD [112]. Prospective, comprehensive and robust studies documenting the prevalence and consequences of these anxiety disorders in patients with CAD are necessary to guide care for patients and orient clinical practice. This data will be very valuable to determine the usefulness of systematic screening for PD and GAD and provide guidance on which aspects of care should be specifically targeted in order to improve the prognosis of this subgroup of patients with CAD.

### Study status

The recruitment began in February 2020. However, the study was on hold from March to July 2020 due to the COVID-19 pandemic confinement measures. Based on the patient volume at the recruitment site, it is estimated that the recruitment target for the present study will be reached in 28 months (30 patients/week). This estimate may be subject to change as the evolution of the COVID-19 pandemic and the associated public health measures may affect the recruitment and total number of eligible patients.

## Data Availability

Not applicable.

## Abbreviations

CAD: Coronary artery disease
GAD: Generalized anxiety disorder
PD: Panic disorder
ADIS-5: Anxiety and Related Disorders Interview Schedule for DSM-5
PTSD: Post-traumatic stress disorder
